# Vascular Smooth Muscle Cell Plasticity in Atherosclerosis: From Macrophage-like Transdifferentiation to Therapeutic Targeting

**DOI:** 10.3390/ijms27209140

**Published:** 2026-10-15

**Authors:** Muxin Zhang, Zicheng Wang, Mengkai Lu, Jiaqi Hao, Yunlun Li

**Affiliations:** 1 First Clinical Medical College, Shandong University of Traditional Chinese Medicine, Jinan 250355, China; muxin.z@hotmail.com (M.Z.); mengkailu0912@163.com (M.L.); goodjq77@163.com (J.H.); 2 Suzhou Institute for Advanced Research, University of Science and Technology of China, Suzhou 215123, China; wizcheng@mail.ustc.edu.cn

**Keywords:** atherosclerosis, vascular smooth muscle cell, phenotypic switching, macrophage-like cell, epigenetic regulation

## Abstract

Atherosclerosis remains the leading cause of cardiovascular mortality worldwide. Vascular smooth muscle cells (VSMCs) were long regarded as passive structural elements of the arterial wall, but they are now recognized as highly plastic cells capable of transdifferentiating into macrophage-like cells (MLCs). This conversion promotes plaque inflammation, matrix degradation, and necrotic core expansion, yet the mechanisms that control it remain poorly defined. This review synthesizes current understanding of the multilayered regulatory networks that govern VSMC-to-MLC transdifferentiation. These networks couple upstream signaling through MAPK, Notch, TGF-β, and PI3K/AKT to master transcription factors such as KLF4, IRF7, and ZEB2. They further extend to epigenetic mechanisms encompassing DNA methylation, histone modifications, non-coding RNAs, and metabolic reprogramming involving cholesterol efflux and lipophagy. We then assess therapeutic strategies that prevent or reverse this conversion, including miRNA replacement, kinase inhibition, LXR activation and autophagy induction. We further highlight unresolved questions on MLC heterogeneity, reversibility, and targeted delivery, outlining priorities for translating these insights into clinical benefit.

## 1. Introduction

Atherosclerosis begins as a chronic inflammatory process within the walls of large and medium arteries, where lipids, cholesterol, and fibrous tissue gradually accumulate in the intima and eventually coalesce into atherosclerotic plaques [1]. The clinical manifestations, including myocardial infarction, angina pectoris, and stroke, arise primarily from thrombotic complications of these plaques. For decades, the dominant hypothesis in atherogenesis research centered on the response-to-injury model, which posited that vascular injury triggers an inflammatory response leading to the recruitment and accumulation of macrophages derived from monocytes [2]. These macrophages were considered the primary architects of the plaque, responsible for engulfing oxidized low-density lipoprotein (oxLDL) to become lipid-laden foam cells, which form the necrotic core of advanced lesions [3]. While this model provided a foundational framework, it failed to fully account for the cellular heterogeneity and the dynamic cellular interactions within the vessel wall.

The application of lineage-tracing technology has reshaped this view. In ApoE^−/−^ mice, SMCs rather than hematopoietic cells contribute the majority of foam cells, with estimates close to 70% in early to mid-stage lesions [4,5]. This proportion is not a fixed value. It varies with disease stage, dietary regimen, and the lineage-tracing strategy used. Human studies support the same conclusion. In human coronary atherosclerosis, co-staining for CD68 and smooth muscle α-actin showed that about 40% of CD68-positive cells originated from SMCs in advanced lesions [6]. Single-cell RNA sequencing later confirmed the presence of VSMC-derived macrophage-like cells (MLCs) [7]. Under resting conditions, medial VSMCs maintain a contractile phenotype essential for vascular homeostasis, expressing a defined set of structural proteins that support arterial tone and wall integrity [8,9]. However, in response to pro-atherogenic stimuli within the plaque microenvironment such as growth factors, inflammatory cytokines, chemokines, lipid accumulation, and hemodynamic stress, VSMCs undergo a profound phenotypic transformation known as phenotypic switching or dedifferentiation [10,11]. This is not a simple binary switch but a transition into a spectrum of diverse and heterogeneous cell states, often termed the synthetic or proliferative phenotype, which is characterized by reduced expression of contractile markers, enhanced migratory and proliferative capacities, and increased secretion of extracellular matrix components and inflammatory mediators [10,12,13]. Some VSMC-derived phenotypes are plaque-stabilizing rather than harmful. Lineage-tracing and single-cell studies have shown that a large fraction of the cells forming the fibrous cap are fibromyocytes, VSMC-derived cells that synthesize collagen and reinforce cap stability [14,15]. In contrast, macrophage-like VSMCs, osteochondrogenic cells, and cells that exhibit impaired efferocytosis are strongly pro-atherogenic and contribute directly to plaque vulnerability through necrotic core expansion, inflammation, and calcification [16]. Among these divergent phenotypes, the VSMC-to-MLC transition is of particular translational relevance.

MLCs originate from VSMCs through dedifferentiation driven by lipid accumulation. They are defined as VSMC-derived cells that simultaneously downregulate contractile genes including Acta2, Tagln, and Myh11, express at least one macrophage marker such as Cd68, Lgals3, Csf1r, or Cd11b within the same cell, and acquire macrophage-like functions such as lipid accumulation, impaired efferocytosis, and extracellular trap formation [17,18]. MLCs do not constitute a single uniform endpoint. VSMCs acquire macrophage features gradually along a continuous path rather than through a one-step conversion between two fixed cell types. Cells traverse a mixed stem cell, endothelial, and monocyte state. Unequal marker loss and gain rates make MLC progression heterogeneous [14,15,19]. The loss of contractile markers and the gain of macrophage markers do not occur at the same rate in every cell, so individual MLCs differ in how far they have progressed. MLCs are enriched in unstable human plaques and their abundance correlates with features of plaque vulnerability. These observations have redefined VSMCs from passive bystanders into active drivers of plaque pathology. Therefore, understanding how VSMCs transdifferentiate into MLCs and the underlying regulatory networks is essential for both basic science and clinical translation. Such knowledge is essential for uncovering the mechanisms underlying atherosclerotic plaque instability and for developing novel therapeutic strategies aimed at stabilizing plaques and preventing acute cardiovascular events.

This review synthesizes current understanding of VSMC-to-MLC transdifferentiation, covering the signaling pathways, transcriptional regulators, epigenetic mechanisms, and metabolic reprogramming that drive this process, and evaluates therapeutic strategies aimed at preventing or reversing phenotype conversion. We conclude by outlining major unresolved questions and future research priorities.

## 2. Characteristics and Functions of VSMC-Derived MLCs

MLCs derived from VSMCs are not simply one cell type or the other. Instead, they are a spectrum of intermediate states that arises when contractile VSMCs lose their differentiated features and acquire a new set of properties normally associated with macrophages. In terms of physical appearance, MLCs are larger than the elongated, spindle-shaped VSMCs from which they originate. Their cytoplasm becomes filled with lipid droplets, giving them a foamy appearance, and their nuclei frequently adopt a lobulated shape reminiscent of macrophages [20,21]. The hybrid identity of MLCs is most clearly reflected in their unusual molecular signature. These cells concurrently express proteins that are traditionally considered lineage-specific for contractile VSMCs, such as α-smooth muscle actin, alongside molecules that are typical of macrophages, including CD68 and Galectin-3 [22]. This dual-positive phenotype is not a culture artifact; it has been consistently observed in situ within atherosclerotic plaques [23]. Lineage-tracing provides the complementary evidence needed to resolve origin. Inducible SMC-specific Cre drivers such as Myh11-CreERT2 activate a heritable reporter in mature VSMCs before lesions develop. The reporter is retained in all progeny even after they lose Myh11, α-SMA, and other conventional SMC markers. A CD68-positive cell that carries the reporter can therefore be assigned to a VSMC origin with certainty. Thus, marker co-expression and lineage-tracing serve complementary roles. Co-staining provides in situ phenotypic evidence and remains the only approach applicable to human specimens, whereas lineage-tracing supplies causal evidence of origin in experimental animals. The two approaches together establish VSMC-to-MLC transdifferentiation as a genuine process [24].

**Figure 1:**
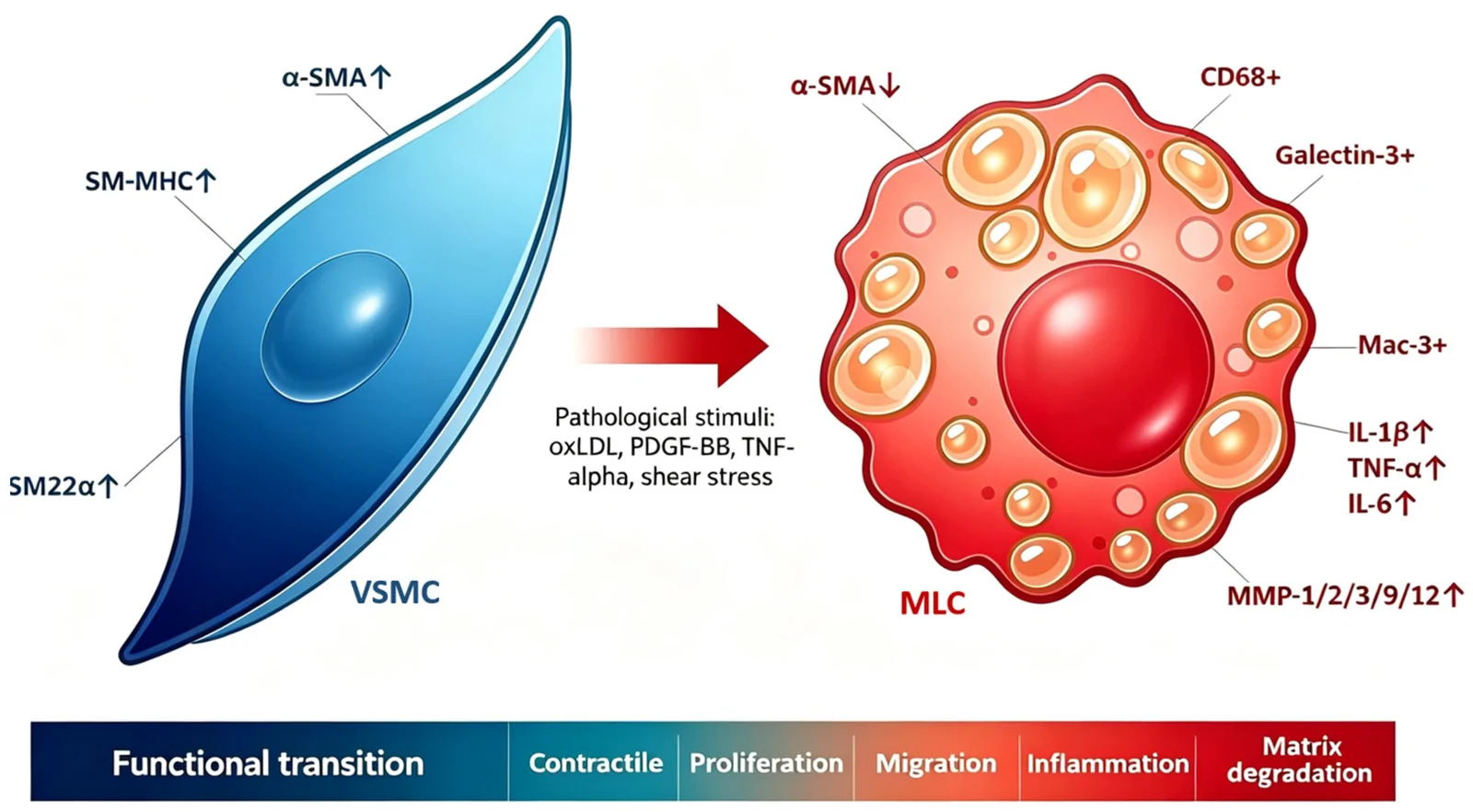
Schematic overview of VSMC-to-MLC transdifferentiation. Pathological stimuli (oxLDL, PDGF-BB, TNF-α, shear stress) drive VSMCs to lose contractile markers (α-SMA, SM-MHC, and SM22α) and gain macrophage markers (CD68, Galectin-3, Mac-3), along with increased production of inflammatory cytokines (IL-1β, TNF-α, and IL-6) and matrix metalloproteinases (MMP-1/2/3/9/12). This functional transition from contraction to proliferation, migration, inflammation, and matrix degradation promotes atherosclerotic plaque vulnerability (↑, increased expression; ↓, decreased expression; α-SMA, α-smooth muscle actin; SM-MHC, smooth muscle myosin heavy chain; SM22α, smooth muscle protein 22 alpha; CD68, Cluster of Differentiation 68; Mac-3, macrophage-3; IL-1β, interleukin 1 beta; TNF-α, tumor necrosis factor alpha; IL-6, interleukin 6; MMP, matrix metalloproteinase; oxLDL, oxidized low-density lipoprotein; PDGF-BB, platelet-derived growth factor BB).

The functional behavior of MLCs derived from VSMCs only partially recapitulates that of classical macrophages derived from monocytes. Firstly, like classical macrophages, MLCs take up and accumulate cholesterol deposited in the intima, forming foam cells laden with lipid. This shift in cellular composition destabilizes the plaque and accelerates the progression of atherosclerotic lesions [25]. Secondly, compared with myeloid macrophages, MLCs display reduced phagocytic and efferocytic capacity. As a result, apoptotic cells accumulate within the lesion and directly promote the formation of the necrotic core [26]. In addition, MLCs mount a vigorous proinflammatory response, with copious secretion of IL-1β, TNF-α, and IL-6 [27]. They also act as potent agents of matrix degradation, releasing a broad panel of metalloproteinases, including MMP-1, MMP-2, MMP-3, MMP-9, and MMP-12. These enzymes digest collagen types I, III, and IV and dismantle the fibrous cap’s supportive scaffold [5,28]. Moreover, activated MLCs in advanced plaques release extracellular traps, which induce collagen degradation and drive surrounding VSMCs toward proinflammatory or senescent phenotypes. These effects exacerbate atherosclerosis and promote progression toward plaque instability [29,30]. As structural integrity erodes and the necrotic core expands, the fibrous cap weakens and becomes prone to rupture under mechanical strain. The plaque is thereby further predisposed to disruption (Figure 1, Table 1).

## 3. Signaling Networks Governing VSMC-to-MLC Transdifferentiation

**Figure 2:**
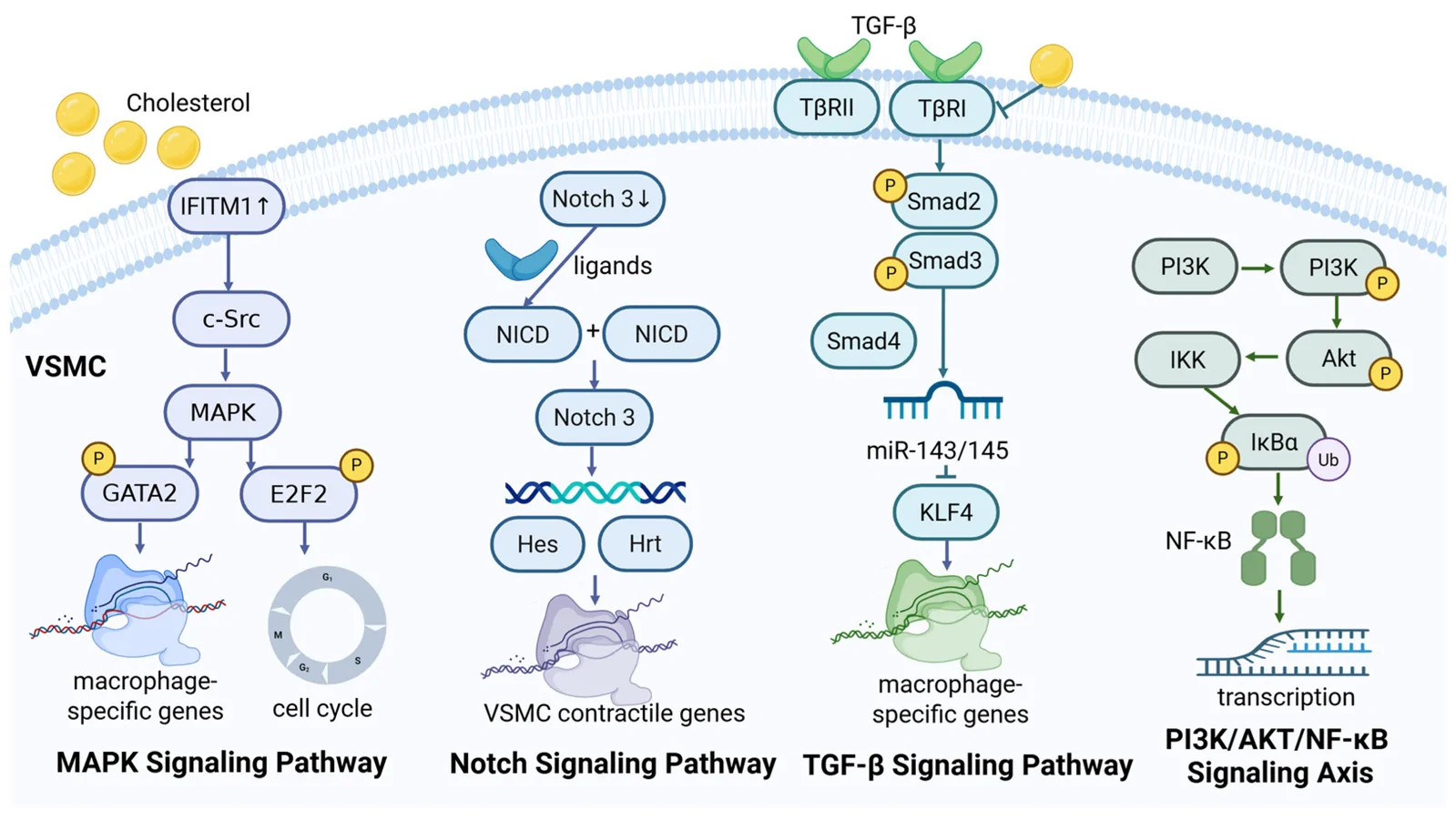
Signaling networks governing VSMC-to-MLC Transdifferentiation (↑, promotion; ↓, inhibition; IFITM1, interferon-induced transmembrane protein 1; c-Src, cellular Src kinase; MAPK, mitogen-activated protein kinase; GATA2, GATA binding protein 2; E2F2, E2F transcription factor 2; NICD, Notch intracellular domain; Hes, hairy and enhancer of split; Hrt, hairy-related transcription factor; TGF-β, transforming growth factor beta; TβRII, transforming growth factor beta receptor type II; TβRI, transforming growth factor beta receptor type I; Smad2, Smad3, and Smad4, mothers against decapentaplegic homolog 2, 3, and 4; miR-143/145, microRNA-143 and microRNA-145; KLF4, Kruppel-like factor 4; PI3K, phosphoinositide 3-kinase; IKK, IκB kinase; Akt, protein kinase B; IκBα, NF-kappa-B inhibitor alpha; Ub, ubiquitin; NF-κB, nuclear factor kappa B. The circled P denotes phosphorylation, and the upward and downward arrows denote increased and decreased expression).

The transdifferentiation of VSMCs into MLCs is a complex process under the precise control of multiple extracellular signals. Several classical signaling pathways, through their aberrant activation or suppression, are the backbone of this regulatory network. These pathways act as central hubs that sense pathological stimuli from the plaque microenvironment and transmit them into the nucleus (Figure 2, Table 2).

### 3.1. MAPK Signaling Pathway: A Membrane-to-Nucleus Cascade Driven by IFITM1 and c-Src

The MAPK signaling pathway, particularly the c-Src/MAPK axis, is a critical driver of VSMC transdifferentiation toward a macrophage-like state [31]. A study established the complete signaling cascade from a cell surface effector protein to transcriptional output. Interferon-inducible transmembrane protein 1 (IFITM1), which is highly upregulated in human atherosclerotic plaques, activates c-Src tyrosine kinase. This activation triggers the MAPK cascade, including p38 MAPK, leading to phosphorylation and activation of downstream transcription factors GATA binding protein 2 (GATA2) and E2F transcription factor 2 (E2F2). These two factors coordinately regulate cell cycle progression and promote a transcriptional program that drives macrophage-associated gene expression, thereby converting VSMCs into MLCs. This mechanism has been independently validated in subsequent studies showing that IFITM1 promotes VSMC lipid accumulation and CD68 upregulation, confirming this pathway as a significant contributor to atherosclerosis [31].

Beyond this IFITM1-mediated model, broader evidence indicates that the MAPK family, including p38, JNK, and ERK1/2, is a general signaling hub for proatherogenic VSMC dedifferentiation [32]. c-Src functions as an upstream general kinase that phosphorylates p38, JNK, and ERK [33,34]. Sustained MAPK activation has been consistently linked to VSMC proliferation, migration, lipid uptake, and macrophage marker expression. This has been demonstrated in cells stimulated with oxidized low-density lipoprotein through the CD36-Src-JNK/p38 pathway [35], as well as in PDGF-BB-induced dedifferentiation models via the Ras/Raf/ERK axis [36].

At the transcriptional level, GATA2 and E2F2 are not merely downstream effectors but form a functional complex. p38-mediated phosphorylation activates GATA2, promoting transcription of macrophage-specific genes, while E2F2 drives cell cycle progression. Their cooperative action is essential for MAPK-mediated MLC generation, as GATA2 knockout completely abolishes this transdifferentiation [31].

Together, these observations depict a regulatory cascade that begins at the cell surface with membrane-bound inducers such as IFITM1 or lipid receptors. The signal then passes through the c-Src/MAPK kinase relay and converges on the GATA2/E2F2 transcriptional complex. This cascade ultimately directs VSMCs toward an unfavorable macrophage-like fate, and its core architecture appears to be evolutionarily conserved.

### 3.2. Notch Signaling Pathway: A Brake on VSMC-to-MLC Transdifferentiation

Studies combining transcriptomics with functional assays show that MLC acquisition is not an all-or-nothing event. Lipid loading alone induces an inflammatory primed state but does not grant full macrophage effector function. Complete reprogramming requires concurrent loss of Notch signaling [37,38,39]. Pharmacological inhibition of Notch using the γ-secretase inhibitor DAPT in cholesterol-loaded VSMCs produces marked gene expression changes and promotes MLC conversion, whereas either intervention alone elicits only limited differentiation-related alterations. Thus, Notch prevents VSMCs from undergoing premature or complete MLC conversion under lipotoxic conditions. Conversely, genetic ablation of RBPJ, the critical downstream effector of Notch signaling, in VSMCs prevents these cells from reacquiring the SMC phenotype within the fibrous cap, further underscoring the necessity of Notch signaling for maintaining VSMC differentiation [40].

At the molecular level, the Notch signaling cascade governing VSMC-to-MLC transdifferentiation involves several sequential events. In normal VSMCs, NOTCH receptors, primarily Notch 3, engage Jagged family ligands expressed by adjacent cells. This engagement triggers sequential proteolytic cleavage by ADAM10/ADAM17 metalloproteinases and γ-secretase, releasing the Notch intracellular domain (NICD). NICD then translocates to the nucleus, where it binds the transcription factor RBPJ and activates downstream target genes including Hes and Hrt families, which maintain VSMC contractile gene expression [41]. Once Notch signaling is suppressed, expression of developmental program genes including Myocd is inhibited, driving VSMCs into a dedifferentiated state characterized by reduced contractile gene expression and progressive upregulation of macrophage-associated genes [37].

### 3.3. TGF-β Signaling Pathway: A Guardian of Contractile Phenotype Suppressed by Cholesterol

TGF-β family members initiate downstream signaling by engaging two types of serine/threonine kinase receptors on the cell surface, type II receptors known as TβRII and type I receptors known as TβRI [42,43]. Upon activation, TβRI phosphorylates Smad2 and Smad3, which then associate with Smad4 and translocate to the nucleus to regulate target gene transcription [44,45]. In healthy vessels, TGF-β signaling preserves the contractile phenotype of VSMCs [46]. In atherosclerotic lesions, however, this signaling is markedly suppressed. Cholesterol overload drives the sequestration of TGF-β receptors into membrane lipid rafts, attenuating signaling efficiency. This suppression dampens miR-143/145 expression, relieving the inhibition on KLF4 and subsequently activating a transcriptional program that upregulates macrophage-related genes while repressing contractile gene expression. The net effect is a progressive conversion of VSMCs toward an MLC phenotype [26,47,48].

Loss- and gain-of-function experiments have validated the significance of this regulatory axis. Partial genetic ablation of Tgfbr2 in VSMCs predisposes these cells to macrophage marker acquisition, a phenotype recapitulated by neutralizing TGF-β antibodies in vivo. Mechanistic relevance to human disease has been confirmed in coronary artery VSMCs. Conversely, high-density lipoprotein-mediated cholesterol efflux reverses receptor sequestration and restores contractile features of cholesterol-loaded VSMCs [47]. These observations establish TGF-β signal suppression as a critical event driving VSMC-to-MLC transdifferentiation in atherosclerosis.

### 3.4. PI3K/AKT/NF-κB Signaling Axis: An Inflammatory Hub Integrating Metabolic and Transcriptional Signals

In the atherosclerotic microenvironment, the PI3K/AKT/NF-κB axis functions as a central hub, linking diverse extracellular pathological stimuli to intracellular pro-inflammatory and pro-transdifferentiation signals. This activation follows a well-defined three-step cascade. The first step involves sensing of upstream signals. Multiple pathological stimuli within the plaque microenvironment can activate this axis, including ox-LDL [49,50], one of the most critical pathogenic factors in atherosclerosis, as well as inflammatory cytokines such as TNF-α and IL-1β [51,52]. Other pathological molecules, including uremic toxin indoxyl sulfate and chemokine-like factor 1, also contribute to its activation [53]. The second step is PI3K/AKT activation and signal amplification. These upstream signals activate PI3K, which subsequently phosphorylates and activates its downstream effector, AKT. Activated AKT functions as a key amplifier within this axis by directly phosphorylating and activating the IκB kinase (IKK) complex in the NF-κB pathway [53]. The third step is NF-κB activation and transcriptional reprogramming. The IKK complex, once activated by AKT, phosphorylates IκBα, the inhibitory protein of NF-κB, marking it for ubiquitination and degradation. Degradation of IκBα liberates the NF-κB dimer, predominantly the p50/p65 heterodimer, allowing its translocation into the nucleus. Once inside the nucleus, NF-κB acts as a transcription factor to initiate transcription of hundreds of target genes, directly driving VSMC transdifferentiation toward the MLC phenotype [50].

Beyond these classical activating stimuli, recent studies have identified several specific regulatory molecules that modulate this signaling axis. HOXA1, a transcription factor, shows markedly elevated expression in the aortic roots of atherosclerotic mice and in diseased human vessels. It drives VSMC-to-MLC conversion through transcriptional activation of NF-κB p65 and KLF4. Knockdown of HOXA1 in ApoE-deficient mice significantly reduces VSMC-to-MLC transdifferentiation, accompanied by suppressed expression of NF-κB p65 and KLF4, positioning HOXA1 as a potential biomarker or therapeutic target [54]. ARF6, a small GTP-binding protein, regulates VSMC phenotypic switching under lipid stimulation. Knockdown of ARF6 reduces VSMC lipid uptake and production of inflammatory cytokines, including IL-6, while simultaneously inhibiting activation of PI3K and NF-κB signaling pathways. In animal models, VSMC-specific ablation of ARF6 markedly reduces atherosclerotic lesion formation and foam cell accumulation [55].

## 4. Transcription Factors Regulating VSMC Transdifferentiation into MLC

**Figure 3:**
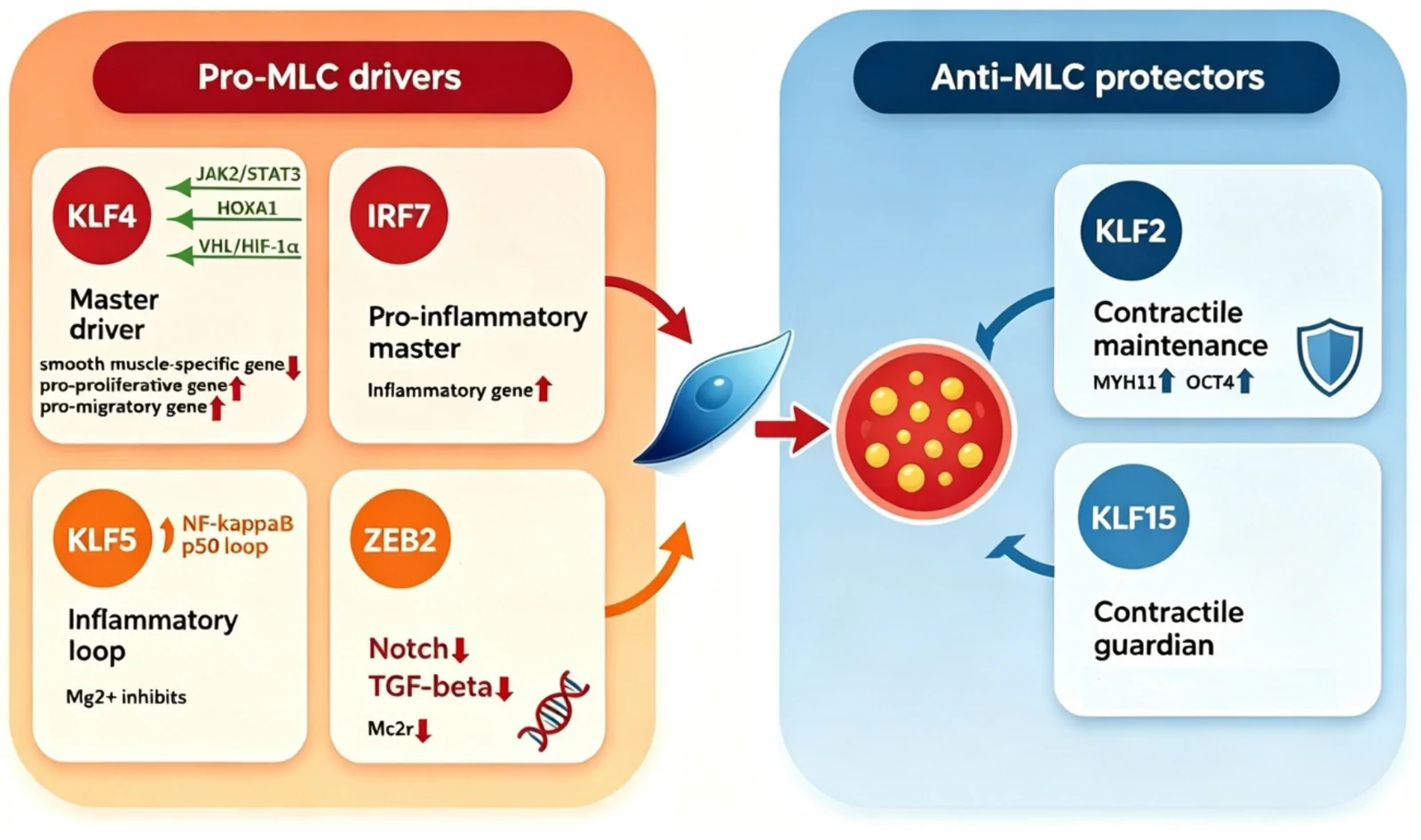
Transcription factors regulating VSMC transdifferentiation into MLC (↑, increased expression; ↓, decreased expression; KLF, Kruppel-like factor; IRF7, interferon regulatory factor 7; ZEB2, zinc finger E-box binding homeobox 2; JAK2, Janus kinase 2; STAT3, signal transducer and activator of transcription 3; HOXA1, homeobox A1; VHL, von Hippel-Lindau tumor suppressor; HIF-1α, hypoxia-inducible factor 1 alpha; NF-κB, nuclear factor kappa B; TGF-β, transforming growth factor beta; Mc2r, melanocortin 2 receptor; MYH11, smooth muscle myosin heavy chain 11; OCT4, octamer-binding transcription factor 4).

The ultimate biological effects of signaling pathways are realized through their regulation of gene transcription. Transcription factors serve as the principal executors that translate upstream signals into specific transcriptional programs (Figure 3, Table 3).

### 4.1. Krüppel-like Factor (KLF) Family

The KLFs are a family of zinc finger-containing transcription factors. Within the cardiovascular system, KLFs are deeply involved in the initiation and progression of atherosclerotic plaques through their regulation of endothelial homeostasis, smooth muscle cell phenotypic switching, and inflammatory signaling [56].

KLF4 functions as a master regulator of VSMC phenotypic plasticity, driving transdifferentiation toward the macrophage-like MLC state in vascular inflammation and atherosclerosis. Cholesterol loading is a key upstream stimulus for KLF4-mediated transdifferentiation. KLF4 integrates signals from multiple upstream pathways, including JAK2/STAT3, HOXA1, and VHL/HIF-1α, to directly drive VSMC transdifferentiation toward the MLC phenotype [57,58]. On one hand, KLF4 cooperates with serum response factor to competitively bind the promoter regions of smooth muscle-specific genes such as SM22α and calponin, thereby suppressing their transcription and leading to the loss of contractile markers [59]. On the other hand, KLF4 collaborates with other transcription factors to activate a program of pro-proliferative and pro-migratory gene expression [60,61]. In vivo evidence from atherosclerotic mouse models demonstrates that conditional knockout of KLF4 reduces VSMC-derived MLCs by more than 60%, confirming KLF4 as a central driver of this transdifferentiation [62].

KLF5 plays a distinct role in VSMC phenotypic regulation compared to KLF4. KLF5 forms a positive feedback loop with NF-κB p50 that sustains the inflammatory phenotype of VSMCs. In inflammatory macrophage-like VSMCs, enhanced nuclear translocation and transcriptional activity of NF-κB p50 upregulate KLF5 expression, which in turn further amplifies NF-κB transcriptional activity, establishing a self-perpetuating inflammatory circuit. Magnesium ion can disrupt this positive feedback loop by inhibiting NF-κB p50 nuclear translocation and consequently reducing KLF5 expression, thereby suppressing the inflammatory phenotype and attenuating MLC formation [63]. In addition, KLF5 participates in flow shear stress-induced VSMC phenotypic switching. Under atheroprone interstitial fluid shear stress, VSMCs undergo synthetic phenotypic conversion through the EGFR/MAPK/KLF5 pathway [64].

KLF2 exerts primarily protective effects in atherosclerosis. It maintains the contractile phenotype by upregulating the expression of genes including MYH11 and OCT4 [62]. KLF2 deficiency leads to a significant increase in atherosclerotic plaque area in ApoE-deficient mice, underscoring its atheroprotective role [65].

KLF15 is highly expressed in VSMCs under physiological conditions but shows markedly reduced expression in atherosclerotic lesions. KLF15 deficiency results in reduced VSMC contractility, exacerbated vascular inflammation, and enhanced extracellular matrix degradation [66].

### 4.2. Interferon Regulatory Factor 7 (IRF7)

IRF7 has recently been identified as a transcription factor that drives VSMC transdifferentiation toward the MLC phenotype [17]. During atherogenesis, VSMCs do not simply convert into a uniform MLC type. Instead, they follow a specific differentiation trajectory. Trajectory inference positions VSMCs along a differentiation pathway that passes through a transitional state with stem cell, endothelial, and monocyte characteristics, ultimately reaching a pro-inflammatory macrophage-like terminal state. Through gene regulatory network analysis, IRF7 was identified as a master transcriptional regulator orchestrating this specific pathogenic transition. Its regulatory activity is significantly higher in pro-inflammatory MLCs than in other macrophage subsets [19].

IRF7 drives VSMC transdifferentiation through multiple mechanisms. Firstly, it orchestrates a comprehensive transcriptional program that directly drives VSMC conversion toward a pro-inflammatory MLC state. Secondly, the IRF7-driven transcriptional program activates a range of inflammation-related genes, endowing cells with potent pro-inflammatory characteristics. Thirdly, within the lipid-rich plaque microenvironment, IRF7 functions as a critical transcriptional checkpoint that sustains and amplifies the pathogenic transdifferentiation of VSMCs toward the pro-inflammatory MLC state [19,69].

Clinical studies have demonstrated that IRF7 expression is significantly upregulated in unstable and advanced human atherosclerotic plaques, correlating strongly with inflammatory macrophage accumulation [19]. In atherosclerotic mouse models, SMC-specific knockdown of IRF7 using an AAV-SM22α-shIRF7 vector significantly attenuated atherosclerotic plaque progression, reduced necrotic core formation, and enhanced fibrous cap stability. These protective effects occurred independently of alterations in lipid levels [19]. The discovery of IRF7 provides new insights for the treatment of atherosclerosis. IRF7 specifically drives pathological VSMC conversion without affecting systemic lipid metabolism, making it an attractive therapeutic target localized within the vascular wall.

### 4.3. Zinc Finger E-Box Binding Homeobox 2 (ZEB2)

ZEB2 is a novel susceptibility gene for atherosclerosis. Genome-wide association studies have identified a signal at chromosome 2q22.3 that is significantly associated with coronary artery disease risk [69,70]. Subsequent CRISPR-Cas9 gene editing experiments confirmed that this risk region corresponds to a long-range enhancer of the *ZEB2* gene. Within atherosclerotic lesions, ZEB2 expression is progressively activated as VSMCs transition from a contractile to a pathological phenotype [69].

The core function of ZEB2 lies in its role as an epigenetic regulatory factor. It alters the epigenetic trajectory of SMCs through chromatin remodeling. Specifically, ZEB2 expression abolishes chromatin accessibility at certain genomic loci, thereby disrupting the normal transduction of two key signaling pathways, Notch and TGF-β [69,71]. Beyond changing cell identity, ZEB2 also profoundly influences metabolic function. Studies have identified ZEB2 as a critical link between VSMC phenotypic switching and dysregulated lipid metabolism. ZEB2 transcriptionally represses melanocortin 2 receptor (Mc2r), constraining the Mc2r-cAMP signaling cascade and downstream fatty acid oxidation programs. This restriction impairs VSMC lipolysis, promotes intracellular lipid accumulation, and accelerates the transition toward a foam cell or macrophage-like state [70].

## 5. Epigenetic Regulation of VSMC Transdifferentiation into MLC

**Figure 4:**
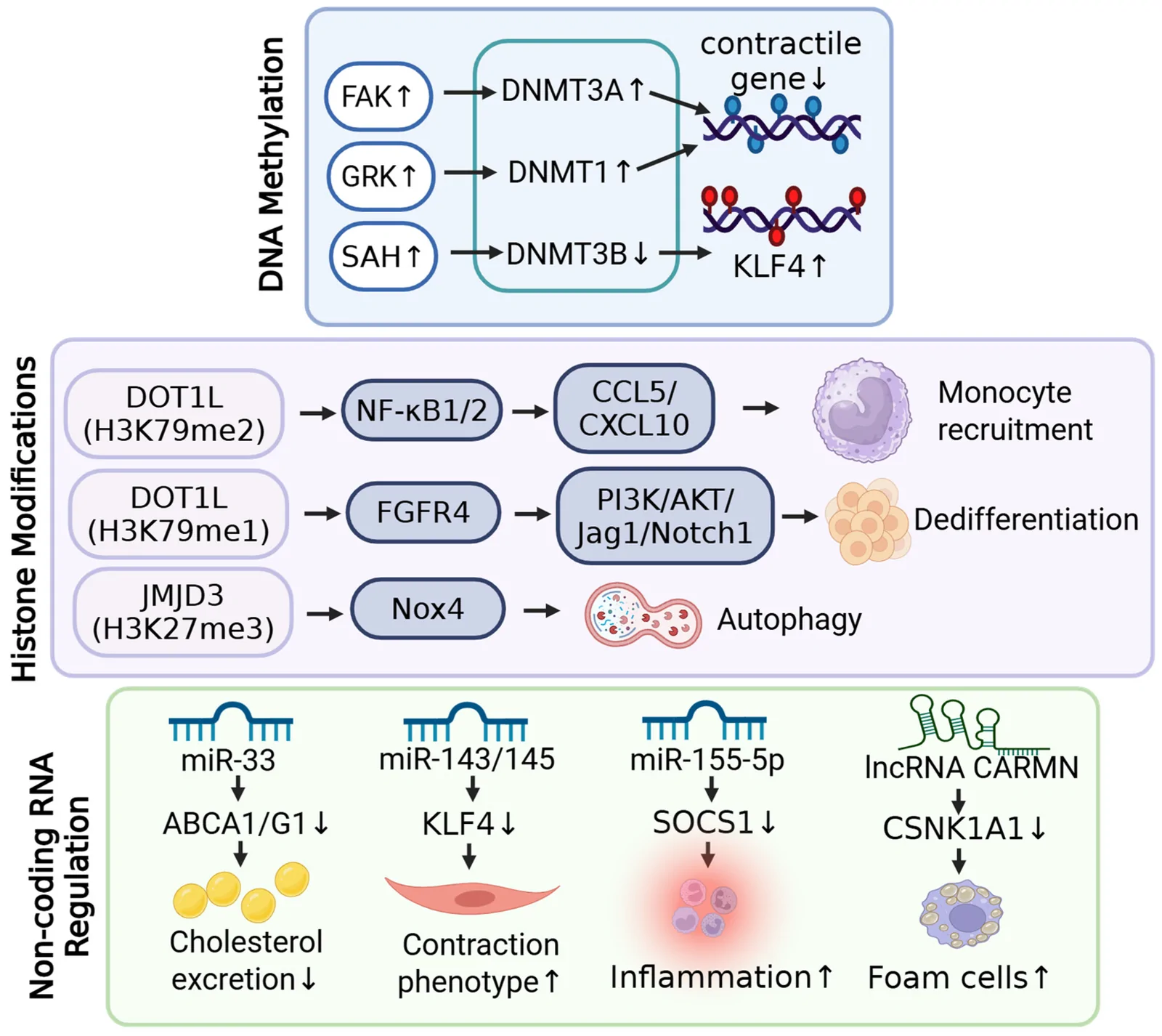
Epigenetic regulation of VSMC transdifferentiation into MLCs (↑, increased; ↓, decreased; FAK, focal adhesion kinase; GRK, G protein-coupled receptor kinase; SAH, S-adenosylhomocysteine; DNMT, DNA methyltransferases; KLF4, Kruppel-like factor 4; DOT1L, disruptor of telomeric silencing 1-like; H3K79me2, histone H3 lysine 79 dimethylation; H3K79me1, histone H3 lysine 79 monomethylation; H3K27me3, histone H3 lysine 27 trimethylation; NF-κB, nuclear factor kappa B; CCL5, C-C motif chemokine ligand 5; CXCL10, C-X-C motif chemokine ligand 10; FGFR4, fibroblast growth factor receptor 4; PI3K, phosphoinositide 3-kinase; AKT, protein kinase B; Jag1, jagged canonical Notch ligand 1; Notch1, notch receptor 1; JMJD3, jumonji domain containing 3; Nox4, NADPH oxidase 4; miR-33, microRNA-33; ABCA1 and ABCG1, ATP-binding cassette transporters A1 and G1; miR, microRNA; SOCS1, suppressor of cytokine signaling 1; lncRNA, long non-coding RNA; CARMN, cardiac mesoderm enhancer-associated non-coding RNA; CSNK1A1, casein kinase 1 alpha 1).

Gene expression is governed not solely by the DNA sequence itself but also by a sophisticated layer of epigenetic modifications. These modifications leave the underlying genetic code intact, yet they modulate gene accessibility and transcriptional activity by reshaping chromatin structure or altering DNA–histone interactions. Epigenetic regulation occurs at three interconnected levels, involving DNA methylation, histone modifications, and noncoding RNAs. Each mechanism contributes unique dynamic features and specificity to VSMC transcriptome reprogramming (Figure 4, Table 4).

### 5.1. DNA Methylation

Epigenome-wide profiling studies have consistently revealed that aberrant DNA hypermethylation within the vessel wall is a defining molecular feature of atherosclerosis [72]. Specifically, DNA isolated from incipient atheromatous lesions carries a heavier methyl load than the corresponding adjacent non-diseased tissue, and the extent of this hypermethylation escalates in parallel with lesion severity [73]. At the level of individual genes, the methylation configuration of promoters driving contractile-marker expression acts as a direct phenotypic switch for VSMCs. In quiescent, contractile VSMCs, the promoters of α-smooth muscle actin, smooth muscle myosin heavy chain, and additional differentiation markers remain relatively unmethylated, keeping chromatin accessible and sustaining robust transcription of these genes. Upon exposure to pathological cues, however, DNA methyltransferases (DNMTs) become upregulated at both the expression and activity levels; the resulting promoter methylation shuts down contractile-gene transcription, constituting one of the earliest and most consequential events in VSMC dedifferentiation. Several lines of experimental evidence support this causal chain. Stabilization of DNMT3A downstream of FAK activation results in hypermethylation of contractile gene promoters, thereby promoting SMC dedifferentiation. Conversely, pharmacological or genetic FAK inhibition drives DNMT3A degradation and promoter hypomethylation, restoring contractile protein expression [74]. An analogous mechanism involves GRK2, a G-protein-coupled receptor kinase that accumulates in dedifferentiating VSMCs. GRK2-mediated phosphorylation protects DNMT1 from degradation, and the stabilized DNMT1 drives promoter hypermethylation and the disappearance of contractile markers; interfering with DNMT1 function reverses this phenotype [75]. The methyl-donor pathway feeds into the same network. Loss of S-adenosylhomocysteine hydrolase causes SAH accumulation, suppresses DNMT3B activity, and thereby lowers methylation at the KLF4 promoter, driving VSMC transdifferentiation toward the MLC phenotype [76]. These findings imply that DNA methylation is reversible. In an unbiased compound screen, the DNMT1 inhibitor 5-azacytidine strongly upregulated PTEN and other SMC differentiation genes [77]. The evidence shows that pathological extracellular signals drive sustained VSMC phenotype switching through dynamic rewriting of promoter methylation.

### 5.2. Histone Modifications

Beyond DNA methylation, histone modifications provide a more complex and plastic layer of epigenetic control. Dysregulation of histone-modifying enzymes drives VSMC phenotypic conversion, exemplified by the H3K79 methyltransferase DOT1L. PDGF-BB upregulates DOT1L in VSMCs, and its expression is confined to VSMCs in atherosclerotic lesions. VSMC-specific Dot1l knockout in ApoE^−/−^ mice reduces atherosclerosis [78]. Combined transcriptomic and H3K79me2 chromatin-profiling analyses show that DOT1L directly regulates Nf-κB1 and Nf-κB2, the master inflammatory regulators. Their activation then induces CCL5 and CXCL10, which recruit monocytes and macrophages to the vessel wall [78]. DOT1L locus variants associate with coronary artery disease. In a diabetic rat model of carotid artery balloon injury, DOT1L engages a distinct route: H3K79me1 deposition at the FGFR4 promoter activates the downstream PI3K/AKT/Jag1/Notch1 axis, which sustains VSMC dedifferentiation [79]. In PDGF-BB-challenged VSMCs, JMJD3 supports cell proliferation and migration and favors the shift toward a synthetic phenotype. In mouse and rat models of carotid injury, loss of JMJD3 suppresses Nox4 expression via H3K27me3 promoter enrichment and reduces neointima formation. Elevated JMJD3 and Nox4 expression has also been confirmed directly in human atherosclerotic plaque specimens [80]. Beyond canonical marks, HDAC3-dependent lactylation mark on H4 lysine 12 has been implicated in VSMC senescence and in atherogenesis [81].

### 5.3. Non-Coding RNA Regulation

Non-coding RNAs regulate VSMC identity. Among them, miR-33, embedded within the *SREBP-2* gene, coordinates cholesterol acquisition and efflux by repressing ABCA1 and ABCG1 [82]. Because these transporters are the main efflux routes in VSMC-derived MLCs, miR-33 directly couples ncRNA activity to foam cell formation. Similarly, cholesterol loading disrupts the miR-143/145–myocardin axis, a core circuit that maintains VSMC contractility, thereby promoting macrophage-like conversion. The miR-143/145 cluster enforces the differentiated state by suppressing KLF4 [83]. In atherosclerotic vessels, reduced miR-145 levels release this brake and permit dedifferentiation. miR-155-5p, by contrast, exerts pro-inflammatory effects through SOCS1 repression in diabetes-accelerated atherosclerosis [84]. Beyond miRNAs, lncRNA CARMN sustains VSMC homeostasis by supporting autophagic flux. Its silencing impairs cholesterol efflux and accelerates foam cell formation through CSNK1A1 repression and subsequent AKT disinhibition [85]. METTL14-dependent m6A modification drives expression of the nuclear receptors PPARα/γ and supports fatty acid β-oxidation, and removing Mettl14 selectively from VSMCs exacerbates disease [86]. Together, miRNAs, lncRNAs, and RNA-modifying enzymes form an interconnected regulatory network that calibrates VSMC transcriptional and metabolic identity during MLC transdifferentiation.

## 6. Metabolism and Lipid Accumulation in VSMC-to-MLC Transdifferentiation

Intracellular lipid accumulation is one of the most conspicuous features of VSMC-to-MLC conversion, endowing these cells with the foam cell morphology that has long been recognized as a pathological hallmark of atherosclerosis. This process is not merely a passive consequence of lipid overload within the plaque. Instead, it actively propels the transdifferentiation process itself [87]. Cellular cholesterol homeostasis is governed by four interconnected processes, including de novo synthesis, uptake, esterification, and efflux. In the atherosclerotic microenvironment, VSMCs are exposed to elevated concentrations of oxLDL. The increased surface expression of scavenger receptors including SR-A and CD36 facilitates unregulated oxLDL internalization, a principal source of lipid accumulation in VSMC-derived foam cells [88].

To prevent cholesterol overload, cells must efficiently export excess sterol. Reverse cholesterol transport fulfills this function by shuttling free cholesterol to extracellular acceptors, and the rate-limiting step of this process, cholesterol efflux, is primarily mediated by the ATP-binding cassette transporters ABCA1 and ABCG1 [87]. ABCA1 preferentially lipidates lipid-poor apolipoprotein A-I to generate nascent high-density lipoprotein particles, whereas ABCG1 mobilizes cholesterol onto mature HDL. Both transporters decline in expression and functional activity as VSMCs transition toward the MLC state. This downregulation drives foam cell formation. A key study addressed the relative contributions of ABCA1 and ABCG1 using CRISPR/Cas9 editing in MOVAS cells to separately ablate each transporter. In cholesterol-loaded ABCA1-null MLCs, apolipoprotein A-I-dependent efflux fell sharply. By contrast, ABCG1-null MLCs retained substantial HDL-dependent efflux. This preservation proved to be due to a compensatory increase in SR-BI, a scavenger receptor that also participates in HDL-associated cholesterol flux. When SR-BI was additionally silenced via short hairpin RNA in ABCG1-null cells, HDL-dependent efflux dropped further [89]. These findings illustrate a marked adaptability in the VSMC efflux machinery, the loss of one pathway can be partially offset by another. Once compensation fails or multiple routes are impaired simultaneously, however, cholesterol accumulates intracellularly and drives pathological MLC conversion.

Pathological regulators that compromise the efflux machinery reinforce this principle. Osteopontin, a matrix glycoprotein enriched in atherosclerotic vessels, engages CD44 on the VSMC surface and dampens p38MAPK-LXRα signaling. This downregulation lowers ABCA1 and ABCG1 expression, diminishing sterol export and promoting foam cell formation [90]. Conversely, enhancing efflux is protective. LXR agonists strongly induce ABCA1 and ABCG1 expression and enhance HDL-dependent cholesterol removal from lipid-laden VSMCs. In vivo, LXR activation reestablishes reverse cholesterol transport from foam-like VSMCs into plasma, HDL, and ultimately feces. Combining an LXR agonist with an ACAT inhibitor, which blocks cholesterol esterification and storage, produces a synergistic restoration of sterol balance [91]. In agreement, selective deletion of LXR in SMCs of hypercholesterolemic mice destabilizes plaques, enlarges necrotic cores and thins fibrous caps while shifting SMC fate toward a more pathogenic trajectory [92].

These plaque-localized mechanisms are significantly amplified by systemic metabolic disease. The metabolic–inflammatory milieu of obesity-related non-alcoholic fatty liver disease (NAFLD) accelerates VSMC-to-MLC transdifferentiation [93]. Excess adiposity and hepatic steatosis elevate circulating IL-6, TNF-α, oxidized lipids, CRP, and MCP-1, which in turn activate the NLRP3 inflammasome and NF-κB [94,95]. These mediators collectively promote VSMC dedifferentiation, macrophage-like transdifferentiation, and extracellular matrix remodeling within the vessel wall. TNF-α drives foam-cell formation through NF-κB, while IL-6 signals via JAK/STAT, thereby linking metabolic disease to plaque progression [96]. NAFLD is accompanied by insulin resistance and atherogenic dyslipidemia, which amplify PI3K/AKT and MAPK signaling. In addition, systemic lipotoxicity and an increased burden of ox-LDL raise the lipid load encountered by VSMCs [97,98,99]. Bile-acid dysregulation, acting through FXR and TGR5 receptors, may further modulate KLF4-dependent transcriptional reprogramming [100]. Therefore, managing NAFLD and its metabolic risk may complement strategies targeting VSMC-to-MLC transdifferentiation.

**Figure 5:**
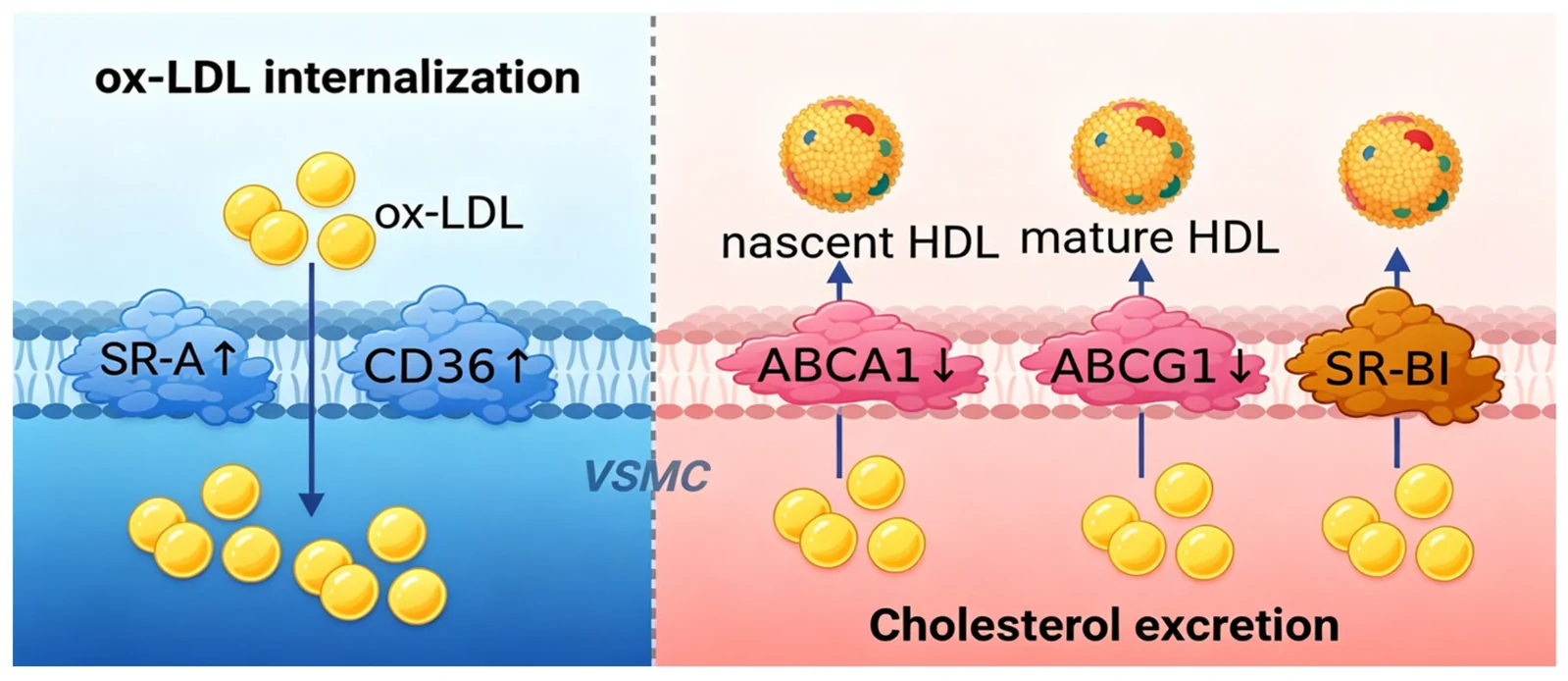
Metabolism and lipid accumulation in VSMC-to-MLC Transdifferentiation (↑, increased; ↓, decreased; oxLDL, oxidized low-density lipoprotein; SR-A, scavenger receptor class A; CD36, cluster of differentiation 36; ABCA1 and ABCG1, ATP-binding cassette transporters A1 and G1; SR-BI, scavenger receptor class B type I; HDL, high-density lipoprotein).

In summary, VSMC fate is determined by the equilibrium between lipid uptake and cholesterol export. When influx exceeds efflux and catabolic capacity, sterol accumulation drives the foam cell and MLC transition, directly linking metabolic dysfunction to plaque vulnerability (Figure 5, Table 5).

## 7. Intercellular Crosstalk in the Vessel Wall

VSMC fate is not determined in isolation. VSMCs are embedded in a multicellular network, and reciprocal signals from endothelial cells, platelets, macrophages, and fibroblasts shape their phenotype. Communication between VSMCs and endothelial cells is bidirectional. Dysfunctional endothelial cells release reduced nitric oxide and increased amounts of endothelin-1, PDGF, TGF-β, and inflammatory cytokines, all of which favor VSMC dedifferentiation [101]. Endothelial cells also signal through direct contact and extracellular vesicles. Endothelial Notch activity, cytokines, and exosomes modulate VSMC phenotype in response to hypoxia, inflammation, and mechanical force. Conversely, extracellular vesicles released by KLF5-overexpressing VSMCs are enriched in miR-155 and induce endothelial injury [102]. Platelets also communicate directly with VSMCs. Activated platelets release PDGF-BB, together with TGF-β, chemokines PF4 and CCL5, thromboxane A2, and platelet-derived microparticles [103]. PDGF-BB is the best-characterized of these signals. It stabilizes the transcription factor Slug and drives VSMC transdifferentiation in rat and human aortic smooth muscle cells, and it acts synergistically with IL-1β to shift human aortic cells from a contractile to a synthetic state [104,105]. VSMCs and macrophages form a self-reinforcing inflammatory loop. VSMCs secrete CCL5 and CXCL10 under DOT1L-dependent control, which recruit monocytes and macrophages into the lesion [106]. Genetic evidence supports the role of CXCL10 because Apoe^−/−^ Cxcl10^−/−^ mice develop smaller lesions with altered T-cell content [107]. Once established, immune cells release PDGF, TNF-α, IL-1β, and TGF-β that further drive VSMC conversion [108]. VSMC-fibroblast interactions operate largely through the shared extracellular matrix. During atherosclerosis, adventitial fibroblasts can transition into myofibroblasts marked by de novo α-SMA expression. These cells proliferate, deposit collagen, and increase matrix stiffness, while altered stiffness feeds back on VSMC lipid handling and phenotype [109].

## 8. Therapeutic Strategies Targeting VSMC-to-MLC Transdifferentiation

Whether VSMC-to-MLC conversion can be pharmacologically halted to stabilize vulnerable plaques is the key therapeutic question raised by the mechanisms outlined above. The therapeutic candidates discussed below are supported almost exclusively by preclinical evidence, including cultured VSMCs; animal models of atherosclerosis; and, in some cases, transcriptomic re-analyses of human plaques. None of these interventions has been validated in clinical trials, and several have not yet advanced beyond proof-of-concept studies.

### 8.1. Preventive Strategies: Maintaining the Contractile Phenotype

As shown in Table 6, preventive strategies aim to intercept the earliest stages of dedifferentiation, preserving the contractile and matrix-synthesizing program before VSMCs commit irreversibly to the MLC fate. These approaches target the signaling, transcriptional, and metabolic nodes that initiate phenotype switching.

At the level of membrane-proximal signaling, the IFITM1/c-Src/MAPK cascade is a rational preventive target. IFITM1 is markedly upregulated in human atherosclerotic plaques and drives VSMC lipid accumulation and macrophage-like conversion through c-Src and downstream MAPK signaling [31]. Small-molecule c-Src inhibitors could, in principle, interrupt this cascade at its apex, although global c-Src inhibition carries off-target risks, and selective targeting of the IFITM1-associated pool would be preferable. The preventive rationale rests on the ability of this pathway to promote lipid accumulation and macrophage-marker expression in VSMCs; no in vivo intervention study has yet quantified MLC abundance after pathway inhibition. G-protein-coupled receptor ChemR23 provides a further preventive entry point. ChemR23 signaling protects against VSMC conversion into macrophage-like foam cells, and its peptide agonist chemerin-9 reduced atherogenesis in ApoE^−/−^ mice [22,110]. This is one of the few preventive candidates whose protective readout directly targets the macrophage-like VSMC fate.

At the transcriptional level, KLF4 is the best-validated master regulator of MLC conversion and a high-priority preventive target. The dietary flavonoid quercetin inhibited the JAK2/STAT3–KLF4 axis in high-fat-fed ApoE^−/−^ mice, reducing lesion size and lipid accumulation. In oxLDL-loaded MOVAS cells, quercetin lowered KLF4 and macrophage markers such as LGALS3, CD68, and F4/80 while restoring α-SMA expression and reducing Dil-oxLDL uptake [57]. Because oxLDL-loaded VSMCs correspond to the foam-cell transition state, the reduction in macrophage markers in this setting provides direct evidence that quercetin can suppress the MLC gene program. IRF7, another driver of the pro-inflammatory MLC state, is a second transcriptional node, although no selective IRF7 modulator has yet been tested in VSMC-specific models. Single-cell transcriptomic re-analysis has additionally identified TNS1 as a driver of contractile VSMC transdifferentiation into macrophage-like SMCs and of plaque destabilization, positioning TNS1 and similar phenotype-determining genes as candidate preventive targets [111].

Non-coding RNA replacement offers an important preventive modality because a single miRNA can coordinate an entire contractile gene network. The miR-143/145 cluster enforces the differentiated state by suppressing KLF4 and other pro-synthetic factors, and its expression declines in atherosclerotic vessels. miR-145 micelles, self-assembling nanoparticles that deliver miR-145 mimics to VSMCs, shifted VSMCs toward a contractile phenotype and mitigated atherosclerosis in ApoE^−/−^ mice [112]. A follow-up study established clinically relevant dosing regimens that curb disease in vivo [113]. In these in vivo studies, miR-145 micelles were administered to mice with established early or mid-stage lesions, and the reported outcomes were reduced plaque growth and reduced pathogenic VSMC populations. Restoration of contractile markers was observed in synthetic VSMCs in vitro, but whether individual MLCs that had already formed in vivo were converted back to a contractile state was not directly demonstrated. The lncRNA ANRIL, induced by metformin, binds the AMPKγ subunit and activates AMPK signaling. Metformin upregulated ANRIL, suppressed PDGF-driven VSMC phenotypic switching, and reduced plaque development in ApoE^−/−^ mice, linking a widely prescribed drug to lncRNA-mediated preventive protection [114]. The metformin evidence reflects inhibition of PDGF-driven synthetic switching rather than reversal of established MLCs.

In addition to these signaling, transcriptional, and non-coding RNA nodes, several interventions prevent MLC formation by acting on lipid handling. Restoration of cholesterol efflux is the most direct metabolic intervention. Pairing an LXR agonist with an ACAT inhibitor, which blocks cholesterol esterification and storage, produces synergistic normalization of sterol balance and prevents the accumulation of lipid-laden VSMC foam cells [91]. Genetic deletion of LXR in SMCs worsens plaque stability, enlarging necrotic cores and thinning fibrous caps, confirming the protective role of this pathway. Systemic LXR activation has been hampered by hepatic steatosis and hypertriglyceridemia, motivating development of LXRβ-selective or VSMC-targeted agonists [92]. On the uptake side, inhibition of AIDA, a scaffold that facilitates CD36 trafficking to the plasma membrane, blunts oxLDL internalization without affecting efflux, thereby limiting the lipid load that drives foam-cell formation [88]. PCSK9 monoclonal antibodies, beyond their LDL-lowering action, reduce VSMC-derived foam-cell formation by downregulating the lncRNA SNHG16, which otherwise scaffolds EZH2 to deposit H3K27me3 at the TRAF5 promoter [115]. A growing number of chemically unrelated compounds further reduce VSMC foam-cell formation by activating autophagy, particularly the selective degradation of lipid droplets known as lipophagy. VSMC-expressed P2RY12 was found to promote VSMC-derived foam-cell formation by suppressing autophagy. P2RY12 inhibition reduced lipid accumulation and VSMC foam-cell formation in ApoE^−/−^ mice independently of LDL cholesterol, including in advanced plaque [116]. Several natural products converge on the same lipophagy axis. Scutellarin, a flavonoid from Erigeron breviscapus, activated autophagy and suppressed NLRP3 inflammasome activation in oleic acid–treated VSMCs [117]. Geniposide, an iridoid glycoside from Gardenia jasminoides, restored lipophagy by inhibiting PARP1/PI3K/AKT signaling [118]. The traditional Chinese medicine herb pair Gualou-Xiebai and its active ingredients suppressed VSMC-derived foam cell formation through P2RY12-mediated lipophagy, a mechanism that aligns with pharmacological P2RY12 blockade [119]. Telmisartan, an angiotensin receptor blocker with partial PPARγ activity, attenuated oxLDL-induced lipid accumulation in VSMCs by inducing autophagy via the PPARγ/AMPK/mTOR pathway, an effect abolished by Atg7 knockdown [120]. It should be noted that these lipid-targeted interventions act on cells that have already accumulated lipid, and their effects were measured as reduced foam-cell formation or reduced lipid content. Whether they can reverse cells that have already acquired macrophage markers has not been established.

### 8.2. Reversal Strategies: Reprogramming Established MLCs

No study to date has formally demonstrated the reversal of established MLCs within atherosclerotic lesions. The available evidence is indirect and falls into three categories. Firstly, the contractile gene program can be reactivated in vitro. miR-145 micelles restored contractile markers, including ACTA2 and MYH11, in synthetic VSMCs derived from patients with varying disease severity, while suppressing KLF4 and KLF5 [112,113]. Pharmacological inhibition of DNA methyltransferases, such as 5-azacytidine, likewise re-induced SMC differentiation genes in cultured smooth muscle cells [77]. These observations show that the contractile program is not permanently lost and can be reactivated, at least in culture. Secondly, the lipid-targeted interventions described in Section 8.1 demonstrate that accumulated lipid can be cleared from foam-like VSMCs through cholesterol efflux or lipophagy. Lipid clearance is likely a necessary step for reversal, but reduced lipid content does not by itself prove that a converted cell has reacquired a contractile identity. Thirdly, no in vivo study has yet combined lineage-tracing with pharmacological intervention to show that VSMC-derived MLCs identified before treatment lose macrophage markers and regain contractile markers after treatment. Until such evidence becomes available, the reversal goal remains a hypothesis supported by indirect evidence.

### 8.3. Translational Challenges and Future Directions

Five interrelated barriers must be overcome before any of these candidates can be evaluated in clinical trials. Firstly, target selectivity and off-target toxicity remain paramount. The pathways driving MLC conversion, including MAPK, Notch, TGF-β, PI3K/AKT, and Wnt, are deeply embedded in normal physiology such as wound healing, vascular development, and immune function. Global inhibition can produce unexpected adverse effects, and strategies that achieve VSMC-specific or pathway-specific modulation will be essential. Secondly, delivery is the single greatest technical obstacle, particularly for nucleic acid therapeutics. Systemically administered siRNAs, miRNA mimics, and antagomirs are rapidly cleared and taken up by the liver and kidney. Achieving therapeutic concentrations in the arterial media, especially within advanced plaques where diffusion is impaired, requires sophisticated carriers such as VSMC-targeted micelles, antibody-drug conjugates, or locally eluting stents. Thirdly, disease and patient heterogeneity demand precision medicine. Atherosclerosis progresses over decades, and plaques vary widely in cellular composition, inflammatory state, and mechanical stability. A therapy that reverses early inflammatory MLCs may be ineffective against late-stage senescent or osteogenic VSMC derivatives. Biomarker-driven stratification combining plasma markers, imaging modalities such as OCT and MRI, and potentially single-cell signatures will be necessary to identify patients most likely to benefit. Fourthly, preclinical model limitations constrain predictive validity. Most evidence derives from hyperlipidemic mouse models and immortalized cell lines. These systems differ from human atherosclerosis in both disease progression and cellular heterogeneity. In terms of progression, mouse plaques are induced by a high-fat or Western diet over weeks to months, whereas human lesions develop over decades. In terms of heterogeneity, the cellular composition, the balance of inflammatory cells, and the spectrum of VSMC transitional states are not fully conserved between mouse and human plaques. Single-cell studies have identified marker and cell-state differences between the two species, and immortalized cell lines further lose the heterogeneity present in primary tissue. Before clinical trials, candidate drugs should therefore be validated in large-animal models, especially porcine and rabbit systems. Human organoids, tissue-engineered vessels, and cross-species single-cell comparison will provide essential support. Fifthly, the temporal window and reversibility of MLC conversion are incompletely understood. Lineage-tracing studies suggest that early MLCs retain plasticity and can revert toward a contractile state, but chronically lipid-laden, senescent, or epigenetically fixed MLCs may be irreversibly committed. Determining whether intervention must occur at specific disease stages and whether combination therapy can unlock late-stage reversal requires longitudinal in vivo studies.

## 9. Materials and Methods

For this review, we performed a comprehensive literature search in PubMed, Web of Science, and Google Scholar for articles published up to 1 September 2026. Search terms covered the full scope of the topic, including “vascular smooth muscle cell”, “VSMC”, “phenotypic switching”, “phenotypic modulation”, “transdifferentiation”, “macrophage-like”, “macrophage phenotype”, “atherosclerosis”, “plaque”, and “metabolic reprogramming”. The reference lists of retrieved articles were additionally screened to identify further relevant studies.

Original research articles and reviews published in English were eligible for inclusion if they addressed VSMC transdifferentiation into MLCs in atherosclerosis. Studies based on lineage-tracing, single-cell sequencing, cultured VSMCs, or models of atherosclerosis were all considered. A small number of studies from vascular injury models were retained as a defined exception when they provided mechanistic evidence directly relevant to atherosclerotic VSMC transdifferentiation. This exception required a disease model that closely mirrors and accelerates atherosclerosis and an explicit statement linking the reported mechanism to atherosclerosis. Conference abstracts, editorials, case reports, articles without full text, and studies published in languages other than English were excluded. Studies without reporting data on the macrophage-like fate were also excluded. An agent-assisted pipeline was used for the search and screening workflow. The agent executed the database queries, deduplicated records, and pre-screened titles and abstracts. Two authors independently verified all outputs and made the final inclusion decisions manually, taking full responsibility for the review. Disagreements were resolved by discussion between the two authors.

## 10. Conclusions

VSMC-to-MLC transdifferentiation overturns the long-held notion that VSMCs are merely passive structural components within atherosclerotic lesions. This transdifferentiation arises from a complex interplay of signaling cascades, transcriptional regulation, epigenetic modifications, and metabolic shifts. The resulting macrophage-like cells drive local inflammation, degrade the extracellular matrix, and expand the necrotic core, thereby promoting plaque vulnerability. Therapeutically, one could either preserve the contractile state or promote a return toward it by enhancing lipid efflux or resetting epigenetic marks. Several existing drugs show promise in preclinical models, although definitive demonstration of MLC reversion remains an open challenge. Future efforts should focus on resolving MLC heterogeneity, defining the temporal window of reversibility, and developing VSMC-selective delivery systems to translate these insights into clinical benefit.

## Figures and Tables

**Table 1:** Comparison of contractile VSMCs and VSMC-derived MLCs.

Feature	VSMC	MLC
Morphology	Spindle-shaped, elongated	Round, foam-cell appearance
Nucleus	Oval, central	Lobulated, eccentric
Lipid droplets	Minimal	Abundant (foamy cytoplasm)
α-SMA/SM-MHC/SM22α	High	Low/absent
CD68/Galectin-3/Mac-3	Negative	Positive
Proliferation/migration	Low	High
Cytokine secretion	Low (homeostatic)	High (IL-1β, TNF-α, IL-6)
Matrix metalloproteinases	Low	High (MMP-1/2/3/9/12)
ECM synthesis	High (collagen, elastin)	Reduced
Functional role in plaque	Fibrous cap stabilization	Promoted inflammation and necrotic core formation

Note: The two columns represent the idealized endpoints of VSMC phenotypic modulation for clarity. In vivo, the transition is continuous and heterogeneous. Individual cells occupy a range of intermediate states in which contractile and macrophage features coexist at varying levels. The listed features should therefore be viewed as trends along a spectrum rather than fixed criteria separating two discrete populations (α-SMA, α-smooth muscle actin; SM-MHC, smooth muscle myosin heavy chain; MMP, matrix metalloproteinase; ECM, extracellular matrix; IL-1β, interleukin 1 beta; TNF-α, tumor necrosis factor alpha).

**Table 2:** Summary of signaling mechanisms, experimental models, and key evidence in VSMC-to-MLC Transdifferentiation.

Mechanism	Experimental Models	Major Evidence	Refs.
IFITM1/c-Src/MAPK	Human atherosclerotic plaques; cultured VSMCs	IFITM1 upregulated in human plaques; activates c-Src → p38 MAPK → GATA2/E2F2 → macrophage gene program; promotes lipid accumulation and CD68 upregulation	[31]
MAPK family (p38/JNK/ERK)	Cultured VSMCs	Sustained MAPK activation links to proliferation, migration, lipid uptake, and macrophage marker expression via CD36-Src-JNK/p38 and Ras/Raf/ERK	[32,33,34,35,36]
GATA2/E2F2 complex	Cultured VSMCs	p38-phosphorylated GATA2 drives macrophage-specific transcription; GATA2 knockout abolishes MLC generation	[31]
Notch signaling	Cholesterol-loaded VSMCs; VSMC-specific RBPJ-knockout mice	Notch loss permits full MLC reprogramming; γ-secretase inhibition promotes MLC conversion; RBPJ ablation prevents SMC phenotype reacquisition in fibrous cap	[37,38,39,40,41]
TGF-β signaling	VSMC-specific Tgfbr2-knockout mice; human coronary artery VSMCs	Cholesterol sequesters TGF-β receptors into lipid rafts; suppression relieves miR-143/145 inhibition of KLF4; Tgfbr2 ablation predisposes to macrophage marker acquisition; HDL-mediated efflux restores signaling and contractile features	[42,43,44,45,46,47,48]
PI3K/AKT/NF-κB axis	Cultured VSMCs	Multiple plaque stimuli (oxLDL, TNF-α, IL-1β, indoxyl sulfate, CKLF1) activate AKT → IKK → IκBα degradation → NF-κB p50/p65 nuclear translocation → pro-inflammatory and pro-transdifferentiation transcription	[49,50,51,52,53]
HOXA1	ApoE^−/−^ mice; human diseased vessels	Elevated in atherosclerotic aortas and human vessels; drives MLC conversion via NF-κB p65 and KLF4; knockdown reduces VSMC-to-MLC transdifferentiation	[54]
ARF6	VSMC-specific knockout mice; cultured VSMCs	Regulates lipid-stimulated phenotype switching; knockdown reduces lipid uptake, IL-6, and PI3K/NF-κB; ablation reduces lesions and foam cell accumulation	[55]

Note: IFITM1, interferon-induced transmembrane protein 1; c-Src, cellular Src kinase; MAPK, mitogen-activated protein kinase; p38, p38 mitogen-activated protein kinase; GATA2, GATA binding protein 2; E2F2, E2F transcription factor 2; CD68, cluster of differentiation 68; JNK, c-Jun N-terminal kinase; ERK, extracellular signal-regulated kinase; CD36, cluster of differentiation 36; Ras, rat sarcoma viral oncogene homolog; Raf, rapidly accelerated fibrosarcoma; Notch, Notch receptor; RBPJ, recombination signal binding protein for immunoglobulin kappa J region; TGF-β, transforming growth factor beta; Tgfbr2, transforming growth factor beta receptor 2; miR-143/145, microRNA-143/145; KLF4, Krüppel-like factor 4; HDL, high-density lipoprotein; PI3K, phosphoinositide 3-kinase; AKT, protein kinase B; NF-κB, nuclear factor kappa-light-chain-enhancer of activated B cells; oxLDL, oxidized low-density lipoprotein; TNF-α, tumor necrosis factor alpha; IL-1β, interleukin-1 beta; CKLF1, chemokine-like factor 1; IKK, IκB kinase; IκBα, nuclear factor of kappa light polypeptide gene enhancer in B-cells inhibitor alpha; HOXA1, homeobox A1; ARF6, ADP-ribosylation factor 6; IL-6, interleukin-6.

**Table 3:** Transcription factors in VSMC-to-MLC transdifferentiation.

Mechanism	Experimental Models	Major Evidence	Refs.
KLF4	Atherosclerotic mice; cultured VSMCs	Master regulator; competes with SRF to silence SM22α and calponin; conditional knockout reduces VSMC-derived MLCs by >60%	[56,57,58,59,60,61,62]
KLF5	Cultured VSMCs;	Forms positive feedback loop with NF-κB p50; Mg^2+^ disrupts the loop and suppresses the inflammatory phenotype; EGFR/MAPK/KLF5 mediates shear-induced synthetic conversion	[63,64]
KLF2	ApoE^−/−^ mice	Upregulates MYH11 and OCT4; deficiency increases plaque area	[62,65]
KLF15	KLF15-deficient mice	Deficiency reduces contractility, increases vascular inflammation and ECM degradation	[66]
IRF7	Human unstable/advanced plaques; ApoE^−/−^ mice; single-cell trajectory and GRN analyses	VSMCs traverse a stem/endothelial/monocyte intermediate to a pro-inflammatory MLC terminal state; SMC-specific knockdown attenuates plaque progression, reduces necrotic core, stabilizes fibrous cap	[17,19,67,68]
ZEB2	GWAS; CRISPR-Cas9-edited cells; human atherosclerotic lesions	2q22.3 risk locus is a long-range ZEB2 enhancer; chromatin remodeling disrupts Notch/TGF-β; represses Mc2r → impaired fatty acid oxidation → lipid accumulation → MLC transition	[69,70,71]

Note: KLF, Krüppel-like factor; SRF, serum response factor; SM22α, smooth muscle protein 22-alpha; NF-κB, nuclear factor kappa-light-chain-enhancer of activated B cells; EGFR, epidermal growth factor receptor; MAPK, mitogen-activated protein kinase; MYH11, myosin heavy chain 11; OCT4, octamer-binding transcription factor 4; ECM, extracellular matrix; IRF7, interferon regulatory factor 7; GRN, gene regulatory network; SMC, smooth muscle cell; ZEB2, zinc finger E-box binding homeobox 2; GWAS, genome-wide association studies; CRISPR-Cas9, clustered regularly interspaced short palindromic repeats and CRISPR-associated protein 9; TGF-β, transforming growth factor beta; Mc2r, melanocortin 2 receptor.

**Table 4:** Epigenetic mechanisms in VSMC-to-MLC transdifferentiation.

Mechanism	Experimental Models	Major Evidence	Refs.
DNA methylation (FAK/DNMT3A)	Human atheroma tissue; cultured VSMCs	Promoter hypermethylation silences contractile genes; FAK stabilizes DNMT3A; FAK inhibition restores contractile protein expression	[72,73,74]
DNMT1/GRK2	Dedifferentiated VSMCs	GRK2 stabilizes DNMT1 → promoter hypermethylation → loss of contractile markers; inhibition reverses phenotype	[75]
Methyl-donor pathway (SAHH/DNMT3B/KLF4)	SAH hydrolase-deficient mice	SAH accumulation suppresses DNMT3B → KLF4 promoter hypomethylation → KLF4 upregulation → phenotype switching and plaque destabilization	[76]
DNMT inhibition (5-azacytidine)	High-throughput compound screen in VSMCs	Upregulates PTEN and SMC differentiation genes	[77]
DOT1L/H3K79me2	ApoE^−/−^ mice (VSMC-specific Dot1l knockout); human genetic variants; VSMC transcriptomic/chromatin profiling	PDGF-BB upregulates DOT1L; knockout reduces atherosclerosis; H3K79me2 directly regulates Nf-κB1/2 → CCL5/CXCL10 → monocyte recruitment; diabetic route via H3K79me1-FGFR4-PI3K/AKT/Jag1/Notch1	[78,79]
JMJD3/H3K27me3	Cultured VSMCs	Promotes proliferation, migration, and synthetic shift; loss suppresses Nox4 via H3K27me3 enrichment, reduces neointima	[80]
HDAC3/H4K12 lactylation	VSMC senescence models	Implicated in VSMC senescence and atherogenesis	[81]
miR-33	VSMC foam cell models	Represses ABCA1/ABCG1 → impaired cholesterol efflux → foam cell formation	[82]
miR-143/145	Cultured VSMCs; human/murine atherosclerotic vessels	Cholesterol disrupts the miR-143/145–myocardin axis → macrophage-like conversion; suppresses KLF4; reduced in lesions	[83]
miR-155-5p	Diabetes-accelerated atherosclerosis models	Exerts pro-inflammatory effects through SOCS1 repression	[84]
lncRNA CARMN	Cultured VSMCs	Supports autophagic flux; silencing impairs efflux, accelerates foam cell formation via CSNK1A1/AKT	[85]
METTL14/m6A	ApoE^−/−^ mice (VSMC-specific Mettl14 knockout)	Drives PPARα/γ expression and fatty acid β-oxidation; deletion exacerbates disease	[86]

Note: FAK, focal adhesion kinase; DNMT3A, DNA methyltransferase 3 alpha; DNMT1, DNA methyltransferase 1; GRK2, G protein-coupled receptor kinase 2; SAHH, S-adenosylhomocysteine hydrolase; SAH, S-adenosylhomocysteine; DNMT3B, DNA methyltransferase 3 beta; KLF4, Krüppel-like factor 4; PTEN, phosphatase and tensin homolog; SMC, smooth muscle cell; DOT1L, DOT1-like histone lysine methyltransferase; H3K79me2, histone H3 lysine 79 dimethylation; PDGF-BB, platelet-derived growth factor-BB; NF-κB, nuclear factor kappa-light-chain-enhancer of activated B cells; CCL5, C-C motif chemokine ligand 5; CXCL10, C-X-C motif chemokine ligand 10; H3K79me1, histone H3 lysine 79 monomethylation; FGFR4, fibroblast growth factor receptor 4; PI3K, phosphoinositide 3-kinase; AKT, protein kinase B; Jag1, Jagged 1; Notch1, Notch receptor 1; EZH2, enhancer of zeste homolog 2; H3K27me3, histone H3 lysine 27 trimethylation; Itgb3, integrin beta 3; JMJD3, Jumonji domain-containing protein 3; Nox4, NADPH oxidase 4; HDAC3, histone deacetylase 3; H4K12, histone H4 lysine 12; miR, microRNA; ABCA1, ATP-binding cassette transporter A1; ABCG1, ATP-binding cassette transporter G1; SOCS1, suppressor of cytokine signaling 1; lncRNA, long non-coding RNA; CARMN, cardiac mesoderm enhancer-associated non-coding RNA; CSNK1A1, casein kinase 1 alpha 1; METTL14, methyltransferase-like 14; m6A, N6-methyladenosine; PPARα/γ, peroxisome proliferator-activated receptor α/γ.

**Table 5:** Metabolic and lipid accumulation mechanisms in VSMC-to-MLC transdifferentiation.

Mechanism	Experimental Models	Major Evidence	Refs.
oxLDL uptake (SR-A/CD36)	Cultured VSMCs	Scavenger receptor upregulation promotes unregulated oxLDL internalization → lipid accumulation and foam cell formation	[87,88]
Cholesterol efflux (ABCA1/ABCG1/SR-BI)	Cultured VSMCs	ABCA1-KO sharply reduces apoAI-dependent efflux; ABCG1-KO partially reduces HDL efflux with compensatory SR-BI upregulation; SR-BI knockdown further reduces efflux	[87,89]
Osteopontin/CD44	Cultured VSMCs	Engages CD44, suppresses p38MAPK-LXRα → ABCA1/ABCG1 downregulation → reduced efflux → foam cell formation	[90]
LXR/ACAT	ApoE^−/−^ mice; SMC-specific LXR-knockout mice; in vivo reverse cholesterol transport assays	LXR activation restores reverse cholesterol transport to plasma/HDL/feces; SMC-specific LXR deletion destabilizes plaques; LXR agonist + ACAT inhibitor synergistically restore sterol balance	[91,92]

Note: oxLDL, oxidized low-density lipoprotein; SR-A, scavenger receptor A; CD36, cluster of differentiation 36; ABCA1, ATP-binding cassette transporter A1; ABCG1, ATP-binding cassette transporter G1; SR-BI, scavenger receptor class B type I; KO, knockout; apoAI, apolipoprotein A-I; HDL, high-density lipoprotein; CD44, cluster of differentiation 44; p38MAPK, p38 mitogen-activated protein kinase; LXRα, liver X receptor alpha; LXR, liver X receptor; ACAT, acyl-CoA:cholesterol acyltransferase.

**Table 6:** Preventive strategies targeting VSMC-to-MLC transdifferentiation.

Drug or Intervention	Target	Mechanism of Action	Experimental Models	Refs.
c-Src inhibitor (candidate)	IFITM1/c-Src/MAPK cascade	Interruption of IFITM1-driven lipid accumulation and macrophage-like conversion	Human atherosclerotic plaques; cultured VSMCs	[31]
Chemerin-9	ChemR23	ChemR23 signaling protects VSMCs from conversion into macrophage-like foam cells	ApoE^−/−^ mice; cultured VSMCs	[22,110]
Quercetin	KLF4	Inhibition of the JAK2/STAT3–KLF4 axis	ApoE^−/−^ mice; MOVAS cells	[57]
TNS1 (candidate target)	TNS1	Unknown, proposed phenotype-determining driver of MLC conversion	Single-cell transcriptomic re-analysis	[111]
miR-145 micelles	miR-143/145 cluster	Restoration of the contractile gene network through suppression of KLF4 and pro-synthetic factors	ApoE^−/−^ mice with early or mid-stage lesions; synthetic VSMCs	[112,113]
Metformin	AMPK (via lncRNA ANRIL)	Upregulation of ANRIL activates AMPK signaling	ApoE^−/−^ mice; cultured VSMCs	[114]
LXR agonist plus ACAT inhibitor	LXR, ACAT	Restoration of cholesterol efflux and blockade of cholesterol esterification and storage	Cultured VSMCs transitioning into foam cells; ApoE^−/−^ mice	[91,92]
AIDA inhibition (candidate)	AIDA/CD36	Blockade of CD36 trafficking to the plasma membrane	ApoE^−/−^ mice; cultured VSMCs	[88]
PCSK9 monoclonal antibody	PCSK9/SNHG16/EZH2	Downregulation of SNHG16 and EZH2-mediated H3K27me3 at the TRAF5 promoter	ApoE^−/−^ mice; cultured VSMCs	[115]
P2RY12 inhibitor	P2RY12	Restores autophagy suppressed by P2RY12 and reduces VSMC foam-cell formation	ApoE^−/−^ mice, including advanced plaques	[116]
Scutellarin	Autophagy/NLRP3	Activation of autophagy and suppression of NLRP3 inflammasome activation	Cultured VSMCs	[117]
Geniposide	PARP1/PI3K/AKT	Restoration of lipophagy via PARP1/PI3K/AKT inhibition	Cultured VSMCs	[118]
Gualou-Xiebai	P2RY12	P2RY12-mediated lipophagy	ApoE^−/−^ mice; cultured VSMCs	[119]
Telmisartan	PPARγ/AMPK/mTOR	Autophagy induction via the PPARγ/AMPK/mTOR pathway	Cultured VSMCs	[120]

Note: All interventions listed in this table are supported by preclinical evidence only. None has been tested in a clinical trial for effects on VSMC-derived MLCs. ACAT, acyl-CoA:cholesterol acyltransferase; AIDA, axin interactor, dorsalization associated; AKT, protein kinase B; AMPK, AMP-activated protein kinase; ANRIL, antisense non-coding RNA in the INK4 locus; CD36, cluster of differentiation 36; ChemR23, chemerin receptor 23; c-Src, cellular Src kinase; EZH2, enhancer of zeste homolog 2; H3K27me3, histone H3 lysine 27 trimethylation; IFITM1, interferon-induced transmembrane protein 1; JAK2, Janus kinase 2; KLF4, Krüppel-like factor 4; lncRNA, long non-coding RNA; LXR, liver X receptor; MAPK, mitogen-activated protein kinase; miR, microRNA; MOVAS, mouse aortic vascular smooth muscle cells; mTOR, mechanistic target of rapamycin; NLRP3, NLR family pyrin domain containing 3; P2RY12, purinergic receptor P2Y12; PARP1, poly (ADP-ribose) polymerase 1; PCSK9, proprotein convertase subtilisin/kexin type 9; PI3K, phosphoinositide 3-kinase; PPARγ, peroxisome proliferator-activated receptor gamma; SNHG16, small nucleolar RNA host gene 16; STAT3, signal transducer and activator of transcription 3; TNS1, tensin 1; TRAF5, TNF receptor associated factor 5.

## Data Availability

No new data were created or analyzed in this study. Data sharing is not applicable to this article.

## Abbreviations

The following abbreviations are used in this manuscript:

VSMCs	Vascular smooth muscle cells
MLCs	Macrophage-like cells
oxLDL	Oxidized low-density lipoprotein
IFITM1	Interferon-inducible transmembrane protein 1
GATA2	GATA binding protein 2
E2F2	E2F transcription factor 2
NICD	Notch intracellular domain
TβRII	Transforming growth factor beta receptor type II
TβRI	Transforming growth factor beta receptor type I
IKK	IκB kinase
KLFs	Krüppel-like factor
IRF7	Interferon regulatory factor 7
ZEB2	Zinc finger E-box binding homeobox 2
Mc2r	Melanocortin 2 receptor
DNMTs	DNA methyltransferases
NAFLD	Non-alcoholic fatty liver disease
α-SMA	α-smooth muscle actin
SM-MHC	Smooth muscle myosin heavy chain
SM22α	Smooth muscle protein 22 alpha
CD68	Cluster of differentiation 68
Mac-3	Macrophage-3
IL-1β	Interleukin 1 beta
TNF-α	Tumor necrosis factor alpha
IL-6	Interleukin 6
MMP	Matrix metalloproteinase
PDGF-BB	Platelet-derived growth factor BB
ECM	Extracellular matrix
c-Src	Cellular Src kinase
MAPK	Mitogen-activated protein kinase
Hes	Hairy and enhancer of split
Hrt	Hairy-related transcription factor
TGF-β	Transforming growth factor beta
Smad2, Smad3, Smad4	Mothers against decapentaplegic homolog 2, 3, and 4
PI3K	Phosphoinositide 3-kinase
Akt	Protein kinase B
IκBα	NF-kappa-B inhibitor alpha
Ub	Ubiquitin
NF-κB	Nuclear factor kappa B
p38	p38 mitogen-activated protein kinase
JNK	c-Jun N-terminal kinase
ERK	Extracellular signal-regulated kinase
CD36	Cluster of differentiation 36
Ras	Rat sarcoma viral oncogene homolog
Raf	Rapidly accelerated fibrosarcoma
Notch	Notch receptor
RBPJ	Recombination signal binding protein for immunoglobulin kappa J region
Tgfbr2	Transforming growth factor beta receptor 2
HDL	High-density lipoprotein
CKLF1	Chemokine-like factor 1
HOXA1	Homeobox A1
ARF6	ADP-ribosylation factor 6
JAK2	Janus kinase 2
STAT3	Signal transducer and activator of transcription 3
VHL	Von Hippel-Lindau tumor suppressor
HIF-1α	Hypoxia-inducible factor 1 alpha
MYH11	Smooth muscle myosin heavy chain 11
OCT4	Octamer-binding transcription factor 4
SRF	Serum response factor
EGFR	Epidermal growth factor receptor
GRN	Gene regulatory network
SMC	Smooth muscle cell
GWAS	Genome-wide association studies
CRISPR-Cas9	Clustered regularly interspaced short palindromic repeats and CRISPR-associated protein 9
FAK	Focal adhesion kinase
GRK	G protein-coupled receptor kinase
SAH	S-adenosylhomocysteine
DNMT	DNA methyltransferases
DOT1L	Disruptor of telomeric silencing 1-like
H3K79me2	Histone H3 lysine 79 dimethylation
H3K79me1	Histone H3 lysine 79 monomethylation
H3K27me3	Histone H3 lysine 27 trimethylation
CCL5	C-C motif chemokine ligand 5
CXCL10	C-X-C motif chemokine ligand 10
FGFR4	Fibroblast growth factor receptor 4
Jag1	Jagged canonical Notch ligand 1
Notch1	Notch receptor 1
JMJD3	Jumonji domain containing 3
Nox4	NADPH oxidase 4
miR-33	MicroRNA-33
ABCA1	ATP-binding cassette transporter A1
ABCG1	ATP-binding cassette transporter G1
miR	MicroRNA
SOCS1	Suppressor of cytokine signaling 1
lncRNA	Long non-coding RNA
CARMN	Cardiac mesoderm enhancer-associated non-coding RNA
CSNK1A1	Casein kinase 1 alpha 1
DNMT3A	DNA methyltransferase 3 alpha
DNMT1	DNA methyltransferase 1
GRK2	G protein-coupled receptor kinase 2
SAHH	S-adenosylhomocysteine hydrolase
DNMT3B	DNA methyltransferase 3 beta
PTEN	Phosphatase and tensin homolog
EZH2	Enhancer of zeste homolog 2
Itgb3	Integrin beta 3
HDAC3	Histone deacetylase 3
H4K12	Histone H4 lysine 12
METTL14	Methyltransferase-like 14
m6A	N6-methyladenosine
PPARα/γ	Peroxisome proliferator-activated receptor α/γ
SR-A	Scavenger receptor class A
SR-BI	Scavenger receptor class B type I
KO	Knockout
apoAI	Apolipoprotein A-I
CD44	Cluster of differentiation 44
p38MAPK	p38 mitogen-activated protein kinase
LXRα	Liver X receptor alpha
LXR	Liver X receptor
ACAT	Acyl-CoA:cholesterol acyltransferase
AIDA	axin interactor, dorsalization associated
AMPK	AMP-activated protein kinase
ANRIL	antisense non-coding RNA in the INK4 locus
ChemR23	chemerin receptor 23
MOVAS	mouse aortic vascular smooth muscle cells
mTOR	mechanistic target of rapamycin
NLRP3	NLR family pyrin domain containing 3
P2RY12	purinergic receptor P2Y12
PARP1	poly (ADP-ribose) polymerase 1
PCSK9	proprotein convertase subtilisin/kexin type 9
SNHG16	small nucleolar RNA host gene 16
TNS1	tensin 1
TRAF5	TNF receptor associated factor 5
